# Anæsthesia and Anæsthetics

**Published:** 1888-05

**Authors:** 


### ANÆSTHESIA AND ANÆSTHETICS. NO. II.

The term anæsthesia primarily means privation of feeling, and an anæsthetic is an agent which produces loss of sensation. In this sense there are very many anæsthetics, not only in drugs, but among physical agents. Cold is an anæsthetic to warm blooded animals, and heat to those of cold blood. A severe shock or a blow upon the head may produce insensibility, or a serious injury to any organ may bring about a local loss of feeling. But in a medical sense, only those drugs which have a specific action upon neural tissues or functions are proper anæsthetics, and the use of the term is usually restricted to a small class of remedies whose action upon other tissues is either comparatively unfelt or brief in its duration. They are divided into general and local agents, according to the extent of the effects produced by them, and the manner of their application. Yet a true anæsthetic always acts in the same general manner, whatever may be the extent of the influence produced by it. Thus we believe that the same general laws apply to cocaine that dominate the influence of chloroform. Both act by a direct impression upon the neural tissue.

A general anæsthetic produces its effects by systemic diffusion. All general remedies must be taken into the circulation, and thus carried to the tissues. To obtain any definite results from a general remedy, enough of it must be existent in the blood at one time, and in whatever manner it is administered the prime object is to get it into the circulatory fluid. This may be accomplished by direct injection into a vein by means of a hypodermic syringe, by rubbing it upon the skin and thus inducing its absorption, by ingestion and the consequent taking up by the lacteals, by enemas and absorption from the intestines, or by inhalation of vapors which are thus taken into the pulmonary circulation, the manner of administration depending largely upon the character of the agent. As anæsthetics are usually of either a gaseous nature or very volatile, they are commonly given by the latter method. There is also the advantage of a direct entrance into the circulation, which is secured in the pulmonary cells, as also the avoidance of gastric complications, which might arise were the agents given by the stomach. A vapor which is breathed has a more direct effect than by any other administration, if we accept direct injection into the circulation. Once in the blood current, the agent is carried to every organ, and to the remotest tissues.

The attempt to determine in what precise way an anæsthetic produces its characteristic effect has so far been a failure. Many experiments have been conducted by the most expert physiologists of which the world has any knowledge, and the main results have been only the multiplication of theories. Claude Bernard believed that he had established the fact that it was by a coagulation of the protoplasmic elements of neural matter. But this has not been accepted by others, who have made a special study of the subject, some of whom believe that in certain agents it is by depriving the tissues of oxygen, while others claim that there is a hyper-oxygenation.

Prof. Anstie believes that it is through a modified blood supply, or by some changes induced in the nutrition of nerve tissues. Whatever the process may be, the result is a modification of nerve function, and this exactly describes what an anæsthetic is, and gives us a general definition of an agent which has a specific power to modify or suspend the transmission of sensory nerve currents. To say that these agents modify or suspend nerve function does not give a perfect definition, for there are drugs which suspend motor nervous impressions without special interference with sensation. Woorari deprives animals of the power of motion, while sensation remains almost or quite intact. In many respects it seems to be the direct antithesis of an anæsthetic, and to produce its effects in a decidedly opposite manner.

A true anæsthetic, then, paralyzes sensor-nervous tissues, or in some way makes them incapable of conducting sensor-nervous impressions. Whether its effects are primarily upon the nerve centers or upon nerve filaments, is not yet determined to the satisfaction of all. To our apprehension the action must be first on the terminal points, for a number of reasons. If it be primarily upon the great ganglia, we should have an influence which would be more nearly instantaneously felt throughout the whole body, and it would paralyze both sensor and motor fasciculi. Its influence would first be manifest upon the nervous centers, and its rate of progression would be far different, for the fact is that the influence of all anæsthetics is from without inward. The first symptoms are manifest and the first effects are felt at the terminal nerve filaments, and the progress is from the extremities toward the ganglionic centers. In this respect it is directly opposite to Woorari, whose primary influence is upon the nervous centers and its progress toward the termini.

The influence or progress of anæsthetic effects could not well be otherwise than from the peripheries toward the nervous centers, from the fact that it is felt only by the sensory nerves, whose office it is to convey, toward the central intelligence, the impressions or undulations received at the outposts. They have no power to transmit impressions from within outward, since the seat of sensation is at the great nervous center. The motor nerves lead outwardly, and are used in response to the information conveyed by the sensor trunks and filaments. If, then, our theory be correct, that the great ganglia, the nervous centers, have for a part of their office the transference of that form of energy which is the result of the molecular changes which we call digestion and nutrition into nerve force, we can better comprehend the physiology of anæsthesia. It is not an arrest of the elimination, but of the conveyance of nervous impulse. It is not of itself fatal, since it does not suspend vitality, only modifying certain manifestations of it. It necessarily causes death only when, having overcome all the outposts, it has cut off the inner citadel from every communication, and is enabled to lay its icy hand upon the involuntary system.

There is another reason why the influence of anæsthetics is from without inward, and it may be found in the fact that it soonest reaches the periphery. It is carried in the blood-current directly to the capillaries, where it first comes in contact with the nerves, the final filaments of which are in immediate connection with the arterioles, and are the first to receive the influence. The progression of sensory impulse has been carefully observed, and its rate been found in man to be about two hundred and fifty feet per second, while in frogs it is but about eighty. It can readily be comprehended, then, that the determination of the progression of the anæsthetic influence may as easily be ascertained as was that of the undulations of nervous impact.

(TO BE CONTINUED.)

